# Pearl Powder—An Emerging Material for Biomedical Applications: A Review

**DOI:** 10.3390/ma14112797

**Published:** 2021-05-24

**Authors:** Xian Jun Loh, David James Young, Hongchen Guo, Liang Tang, Yunlong Wu, Guorui Zhang, Changming Tang, Huajun Ruan

**Affiliations:** 1 Institute of Materials Research and Engineering, Agency for Science, Technology and Research, 2 Fusionopolis Way, Singapore 138634, Singapore; 2 College of Engineering, Information Technology and Environment, Charles Darwin University, Darwin, NT 0909, Australia; david.young@cdu.edu.au; 3 Zhejiang Fenix Health Science and Technology Co., Ltd., Zhejiang 176849, China; jessenghc@hotmail.com (H.G.); TangL_1234562021@163.com (L.T.); zgr77129219@163.com (G.Z.); tang1chang2ming@163.com (C.T.); 4 Research State Key Laboratory of Cellular Stress Biology, Fujian Provincial Key Laboratory of Innovative Drug Target, School of Pharmaceutical Sciences, Xiamen University, Xiamen 361102, China; wuyl@xmu.edu.cn

**Keywords:** pearl, nacre, skin treatment, wound healing, bone repairing

## Abstract

Pearl powder is a well-known traditional Chinese medicine for a variety of indications from beauty care to healthcare. While used for over a thousand years, there has yet to be an in-depth understanding and review in this area. The use of pearl powder is particularly growing in the biomedical area with various benefits reported due to the active ingredients within the pearl matrix itself. In this review, we focus on the emerging biomedical applications of pearl powder, touching on applications of pearl powder in wound healing, bone repairing, treatment of skin conditions, and other health indications.

## 1. Introduction

Pearl powder has been used for more than a thousand years for traditional Chinese medicine, cosmetics, and as a health food substitute [1,2]. It is dense in protein and mineral content and has been used to treat a variety of skin and bone disorders, as well as palpitations, insomnia, and epilepsy [3,4]. The pearl-farming industry originated in Japan over one hundred years ago. Today, pearls are harvested worldwide for jewellery and also for their putative health benefits [5]. Additionally, pearls have been widely used as cosmetic agents and the utilization of pearl powder for maquillages can be traced back to as early as the Northern Song Dynasty. One of the key archaeological sites for pearl powder-based cosmetics in China is the family tombs of Lu Dalin in Shaanxi Province. A porcelain box discovered in the tombs, known to be a woman’s lipstick, containing white powder which matched the material characteristics of high-quality freshwater pearls [6]. At present, pearls are cultured on a large scale in many countries; and so while the price of perfectly spherical, lustrous pearls for jewellery can be very high, the price of pearl powder for health care and cosmetic products is comparatively low.

Calcium carbonate (CaCO_3_) and magnesium carbonate are the primary ingredients of mollusc shells, with the remaining organic matrix containing proteins, glycoproteins, and polysaccharides [7]. The rest of the shell includes silica, calcium phosphate (Ca_3_(PO_4_)_2_), aluminium oxide, and iron oxide. Pearl also contains trace elements like sodium, manganese, selenium, aluminium, and copper. Animal studies have demonstrated that pearl powder has beneficial pharmacological effects such as anti-oxidant, anti-inflammatory (due to the magnesium that it possesses), anti-aging (through the stimulation of fibroblasts), immunomodulating, and wound healing [3,8,9,10,11]. As such, pearl powder has been integrated into health foods and used to treat ulcers (e.g., aphthous, gastric, and duodenal) [4,9]. Essential amino acids like aspartate (Asp) and glutamate (Glu) are also present, possessing antioxidant properties that could boost up the immune system [12,13,14,15]. Hydrophobic amino acids such as cysteine (Cys), isoleucine (Ile), leucine (Leu), methionine (Met), phenylalanine (Phe), tryptophan (Trp), and valine (Val) also possess good free radical quenching ability [16], further enhancing the antioxidant properties of pearl powder. The presence of calcium, magnesium, and selenium endow pearl powder with antioxidant activity by acting as cofactors to antioxidant enzymes [17]. The iridescent inner shell of certain molluscs (mother of pearl) and pearls are composed of nacre—an inorganic/organic composite composed mainly of CaCO_3_ in the aragonite isomorph. Bones, on the other hand, consist mainly of Ca_3_(PO_4_)_2_ and hydroxyapatite (HA). Both exist naturally as composites via biomineralization and have a structural supporting function [18,19]. Pearl contains more protein and trace metal ions than shell nacre, which contributes to its potential biomedical applications such as stimulating bone growth.

**Figure 1:**
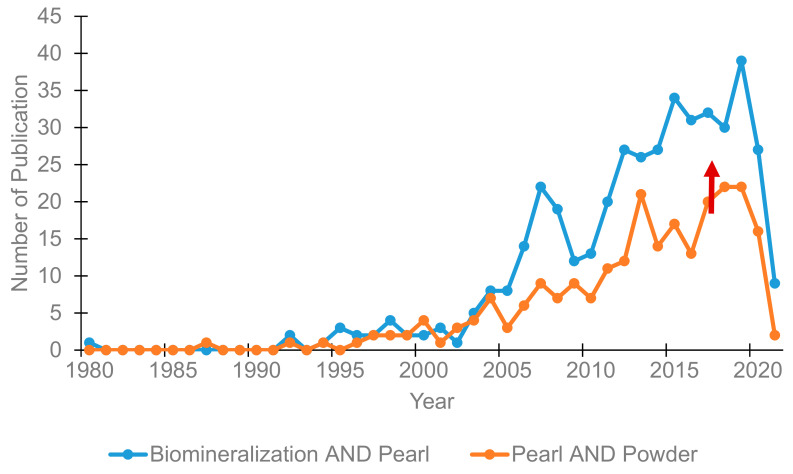
Number of publications per year from the Web of Science search for articles with topics of search terms “Biomineralization AND Pearl” and “Pearl AND Powder”, dated 16 April 2021.

This review focuses on recent advances made in the use of pearl powder for biomedical applications, including wound healing, tissue engineering, and bone regeneration. Of note, we wish to highlight that the biochemistry and application of pearl powder are relatively nascent interests that are set to grow. The number of research papers in the study of biomineralization of pearls has risen steadily over the last 10 years (Figure 1). It is only in recent years that pearl powder has been scientifically investigated for various benefits. The most common application work of pearl powder is focused on wound healing and bone regeneration. While the number of publications on pearl powder is still small, we expect the number of publications to grow further in the coming years. With this in mind, we summarise recent findings on pearl powder with an in-depth look at the issues and potentials. 

## 2. Biomineralization in Pearls

Calcium carbonate (CaCO_3_), one of the most abundant minerals on earth, is the main component in pearls, i.e., the nacre of mollusc shells and otoliths. Anhydrous CaCO_3_ can exist in three polymorphs depending on environmental conditions: calcite, aragonite, and vaterite, of which calcite is the most thermodynamically stable, followed by aragonite and vaterite [20,21]. These three polymorphs appear as single crystalline cubes, needle-like crystals, and polycrystalline spherulites, respectively [22]. Aragonite and calcite are commonly found in nature while vaterite requires extreme conditions with a specific combination of pH, temperature, and pressure to form [23,24]. Vaterite can also be prepared in the laboratory by employing organic additives or templates. However, vaterite is unstable in an aqueous solution, in which it can undergo phase transformation to calcite within 20–25 h at room temperature. At a higher temperature (~60 °C), vaterite can rapidly transform to aragonite [25,26]. 

Most freshwater pearls have lustre. Aragonite is the main inorganic component that contributes to the lustrous property of pearls, giving rise to the term “aragonite pearls” [27,28]. Vaterite is mainly found in half-lacklustre or lacklustre pearls as a result of irregular CaCO_3_ biomineralization during the formation of pearls, giving rise to the term “vaterite pearls” [28]. As mentioned above, nacre is a term to describe the natural biocomposite formed from CaCO_3_ biomineralization, consisting of aragonite (ca 95 wt.%), matrix proteins and other soft organic biopolymers (ca 5 wt.%). Crystallization of CaCO_3_ is primarily stimulated by the organic matrix in the nacreous layer. Various organic matrices had been extracted and purified from the nacreous layer of mollusc shells using ethylenediaminetetraacetic acid (EDTA) or acetic acid, and were subsequently tested for in-vitro CaCO_3_ crystallization to clarify the mechanism of biomineralization in nacre [29,30,31,32,33]. The matrix plays important roles in determining the nucleation site, crystal size, and morphology of CaCO_3_ crystals. Gong et al. have identified a nacre matrix protein originated from the mantle epithelia of *Pinctada fucata* pearl that initiates crystal nucleation and calcium deposition with inconsistent morphology [34]. Zhang et al. have also identified a nacre matrix protein from *P. fucata* that contributes significantly in controlling the crystallization of CaCO_3_ and inducing the formation of needle-like aragonite crystals [35]. Most of these studies have concentrated on the role of proteins, but there is a lack of information on the mechanism and dynamics of biomineralization. 

Decalcification by disodium ethylenediaminetetraacetate dihydrate (Na_2_EDTA) is the primary extraction method of nacre organic matrix, but this method potentially undermines the structures or roles of proteins by sequestering essential metal ions. To overcome this, weak acid can be used to extract organic matrices from pearls [36]. Aizenberg et al. first introduced the gas diffusion method to examine how soluble organic matrix affects the crystallization of CaCO_3_ [37]. This method mimics in vivo crystallization condition in which it delivers supersaturated CaCO_3_ solutions gradually and continually. To characterize the crystals, scanning electron microscopy (SEM) is utilized to observe the morphologies and Raman spectroscopy has also been proven to be a powerful technique to distinguish the polymorphs of CaCO_3_. Progress has been made using infrared reflectance spectroscopy as well. Mixtures of pearl powder and shell powder were calcined at various temperatures for different periods followed by analysis by infrared reflectance spectroscopy [38]. When calcined at 400 °C for 30 min, aragonite in pearl powder was partially transformed into calcite, while aragonite in shell powder was totally transformed into calcite, thus differentiating between the two sources. The difference in phase transition between pearl powders and shell powders can then be deduced from infrared reflectance spectroscopy. Another effective method with enhanced resolution based on Tri-step infrared spectroscopy coupled with chemometrics has also been developed for qualitative classification of pearl powders according to pearl contents and quantitative analysis of shell powders in adulterated pearl powders [39]. Apart from the aforementioned techniques, computer vision technology (E-eyes) has also been employed to categorize pearl powders into trait-visual colour groups through colour measurement. Although different grades of pearl powder have similar IR spectral characteristics, higher intensity of second derivative infrared spectra (SD-IR) peak at around 861 cm^−1^ represents better consistency of pearls, while intense peaks at 1082 cm^−1^, 712 cm^−1^, and 699 cm^−1^ showed the opposite. Bands in ranges 1550–1350 cm^−1^, 730–680 cm^−1^, 830–880 cm^−1^, and 690–725 cm^−1^ were also useful for distinguishing pearl powders and recognizing adulteration.

## 3. Applications

### 3.1. Wound Healing and Treatment of Skin Conditions 

Acute injuries, including burns and ulcers, are complex lesions that can be difficult to treat. Wound healing occurs in three phases, viz inflammation, proliferation, and maturation [40]. Inflammation occurs as a result of the release of elastases and proteases by neutrophils with vascular dilation and increased blood vessel permeability. During the second proliferation phase, migration of keratinocytes to the injured dermis takes place to promote wound angiogenesis, in which new blood vessels form in the damaged tissue. Some fibroblasts would differentiate to myofibroblasts and together they produce the collagen extracellular matrix (ECM) in the third maturation stage [10,41]. Wound healing can be affected by hypoxia, bacterial infection, poor local blood supply, age, and comorbidities such as diabetes [42,43,44]. Unfortunately, there are very few therapeutic agents available for stimulating wound healing. The only product approved by the Food and Drug Administration (FDA) is platelet-derived growth factor (PDGF)-BB although caution is recommended because of possible promotion of tumours [45]. There are active components in pearl powder that are beneficial for skin cell regeneration and therefore valuable for wound healing. Mother of pearl (nacre) (*Pinctada maxima*) was implanted into rat dermis and demonstrated that it fostered better skin tone while stimulating proper physiological functioning in skin fibroblast [46], having promising potential for skin regeneration. In brief, implanted nacre cultivated well-vascularized tissue and improved ECM production; increased synthesis of substances involved in cell adhesion and communication (e.g., decorine) and tissue regeneration (e.g., collagen types I and III). Besides nacre, pearl extract was also demonstrated to be able to promote fibroblast migration in an in vitro wound healing model by human fibroblast cells [9]. Medium-containing PL (300 µg/mL) increased migrating fibroblast cell numbers by three times relative to a control without PL. Moreover, PL containing medium stimulated mRNA expression of collagen type III in fibroblasts, boosting wound healing. 

**Figure 2:**
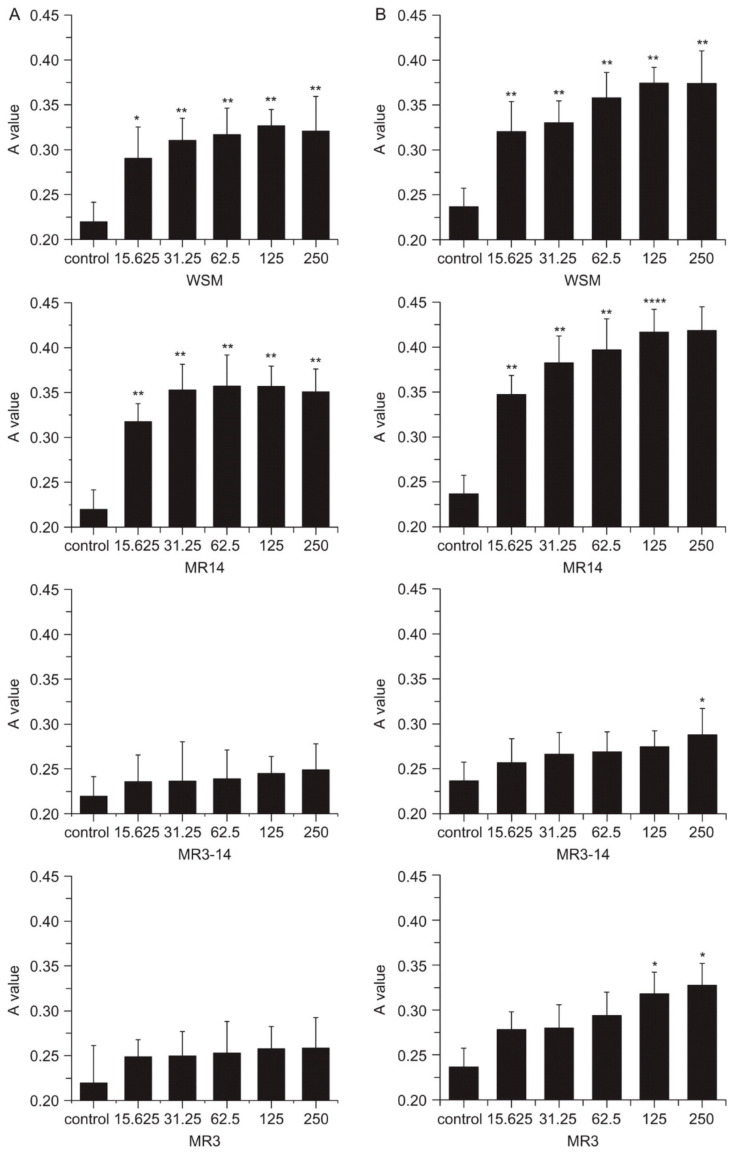
The water-soluble matrix (WSM) was extracted from pearl powder and size-fractionated using a 14 kDa cut-off dialysis tubing to give >14 kDa retentate (MR14), followed by a 3 kDa tubing dialysis to give 3–14 kDa retentate (MR3-14) and <3 kDa retentate (MR3). Effects of WSM, MR14, MR3–14, and MR3 on the growth of fibroblasts (μg/mL): incubation (**A**) after 2 days and (**B**) after 3 days. Reproduced from [49]. * *p* < 0.05; ** *p* < 0.01; **** *p* < 0.005.

To further understand the role of active components of nacre on skin therapies, Rousseau et al. isolated lipids from the nacre of *Pinctada margaritifera* and tested its effect on artificially dehydrated skin explants as a representation for atopic dermatitis [47]. Nacre lipids were found to facilitate the reconstitution of intercellular content of the stratum corneum and could be responsible for the signalling action activated for treating atopic dermatitis. The beneficial effect of pearl powder may come from conchiolin, a protein made of 17 amino acids [48]. In the beauty world, it is claimed that conchiolin hydrates the skin and rebuilds collagen. As such, a hypothesis on wound healing properties of pearl powder is made—conchiolin is the main contributor to the excellent wound healing properties of pearl powders by coupling essential minerals. This is, however, unsubstantiated and needs to be scientifically verified. Lee et al. reported the impact of water-soluble components of mother of pearl (nacre) on second-degree burns of porcine skin as a surrogate for human skin [8]. The application of water-soluble components of nacre triggered burn-induced granulation areas to quickly fill with collagen with a natural skin appearance reforming on the wounded dermis and epidermis. Angiogenesis and regeneration of wounds in apoptotic and necrotic cell injuries were promoted by water-soluble components of nacre. This is further supported by the murine fibroblast NIH3T3 cells model treated with water-soluble components of nacre, which showed improved proliferation and collagen production. Compared to powdered nacre, water-soluble components of nacre possessed better inherent biocompatibility, having more promising potential for wound healing. Jian-Ping et al. employed a mouse model to demonstrate that the water-soluble matrix (WSM) of pearl powder (*Hyriopsis cumingii*) could induce oral fibroblast proliferation and collagen aggregation, suppress the activity of matrix metalloproteinase-2, and significantly promote TIMP-1 synthesis, benefiting wound healing [49]. Different sizes of water-soluble protein fractions were obtained and a fibroblast growth study indicated the larger molecular weight components had a greater influence on fibroblast proliferation (Figure 2). 

**Figure 3:**
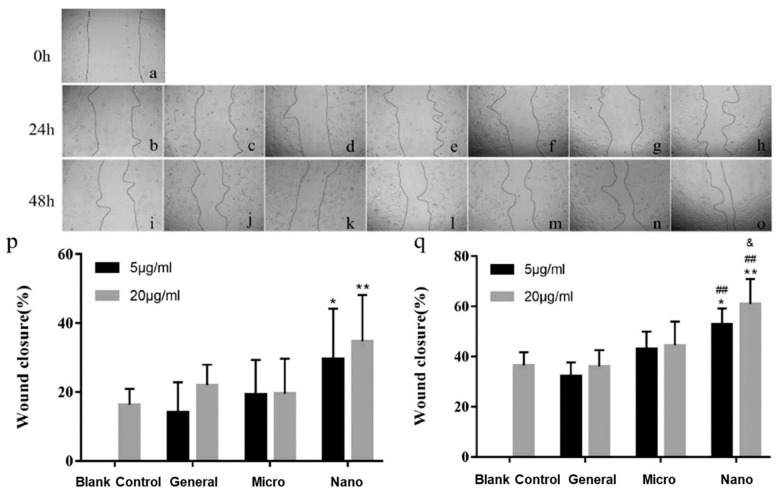
Different concentration and particle sizes of pearl powder and their effects on keratinocyte migration: (**a**) injury at 0 h; (**b**,**i**) control group; (**c**,**j**) pearl powders with concentration of 5 μg/mL; (**d**,**k**) pearl powders with concentration of 20 μg/mL; (**e**,**l**) micron-sized pearl powders with concentration of 5 μg/mL; (**f**,**m**) micron-sized pearl powders with concentration of 20 μg/mL; (**g**,**n**) nano-sized pearl powders with concentration of 5 μg/mL; (**h**,**o**) nano-sized pearl powders with concentration of 20 μg/mL. Statistical data of injury closure percentage in wound scratch assay in (**p**) 1 day and (**q**) 2 days, determined by Image J software (U.S. National Institutes of Health, Bethesda, MD, USA, v1.51 23 April 2018). Reproduced from [4]. (* *p* < 0.05, vs. blank control; ** *p* < 0.01, vs. blank control; ## *p* < 0.01, vs. general; & *p* < 0.05, vs. micro).

Nanotechnology holds promise for revolutionizing approaches for tackling issues by unravelling the possibilities at a nanoscale level. Chen et al. have investigated pearl powders of various particle sizes for wound care using the rat skin excision model [4]. Both micro and nano-sized pearl powders enhanced the distribution and passage of skin cells and hastened wound closure, as well as greatly improved the biomechanical strength of the recovered tissue. Treatment with pearl powder could increase the formation and daily deposition of collagen and promote angiogenesis of the skin. Nano-sized pearl powder exhibited the highest healing efficacy. This is likely to be attributed to the relatively larger surface areas of nano-sized pearl powders and the amount of water-soluble protein that can be extracted is greater than that of coarser powders. In addition, nano-sized particles can access the site better than larger particles by mechanisms such as endocytosis and active membrane transport (Figure 3). On another note, nano-sized water-soluble pearl powder (NWSNP) facilitated the differentiation of MC3T3-E1 preosteoblast cells by enhancing autophagy through the mitogen-activated protein kinase (MEK)/extracellular signal-controlled kinase (ERK) signalling pathway [50]. NWSNP significantly increased the expression of autophagy markers microtubule-associated light chain 3 (LC3)II/I, Beclin-I, and autophagy-associated 7 (ATG7). MTT assay revealed that the viability of MC3T3-E1 preosteoblast cells was at its best with the highest dose of WSNNP used in the study (50 mu g/mL). In a dose-dependent manner, WSNNP significantly increased the amount of differentiation markers type 1 collagen (COL-1), runt-related transcription factor 2 (RUNX2), secreted phosphoprotein l (SPP1), and alkaline phosphatase (ALP). 

The advancement of antimicrobial nanofibre dressings that provide protection to the injured tissues from commensal pathogens while promoting tissue regeneration at the same time would be significant in plastic and reconstructive surgery practices. Recently, Mayandi et al. studied the effect of chondroitin sulphate on the physicochemical properties of polydopamine crosslinked electrospun gelatin nanofibres containing mineralized magnesium [44]. Composite dressings comprising polycaprolactone (PCL) and gelatin were also prepared to lengthen the durability of the dressings. These dressings showed enhanced mechanical properties and flexibility, while core–shell nanofibres exhibited superior photoluminescent properties. Most importantly, these dressings displayed enhanced re-epithelialization, wound closure, and clinical outcome in a porcine model of cutaneous burn injury compared to untreated burns. Histology of the biopsied tissues revealed a smooth regeneration and organization of collagen on the burns treated with core–shell nanostructures. This study has shown that mineralized composite nanofibres are capable of accelerating burn wound healing and exhibit antimicrobial action, underlining their potentials as wound dressings and skin substitutes.

### 3.2. Anti-Fibrotic and Anti-Inflammatory Action

Yang et al. used a room-temperature super-extraction method to generate the key active components of pearl and mixed the pearl extracts with poly(gamma-glutamic acid) hydrogels. The hydrogels with pearl extracts were effective in enhancing anti-inflammatory and anti-apoptotic function in human keratinocyte (HaCaT) cells exposed to low-dose ultraviolet B (UVB) [51], suggesting a marketed pearl extract could be capable of preventing radiation dermatitis in keratinocytes. Latire et al. used a primary culture of human dermal fibroblasts to determine the possible biological function of matrix macromolecular components derived from the shells of edible molluscs (the blue mussel *Mytilus edulis* and the Pacific oyster *Crassostrea gigas*) [7]. Both extracts had beneficial effects on cell metabolic function. Both treatments decreased amounts of COL-1, together with an improvement in the activity of matrix metalloproteinase-1, suggesting that both extracts have promising potential for use in the treatment of fibrosis, in particular, for scleroderma.

### 3.3. Antioxidant and Anti-Aging Applications

Lipid peroxidation can be induced by free radical oxidation and cause irreversible impairment of cellular macromolecules such as membrane lipids, proteins, and nucleic acids via reactive oxygen species (ROS) [14]. In brief, excessive production of ROS will lead to cellular injury and expedite the process of aging. An imbalance between ROS and antioxidant causes oxidative stress. Continuous ROS production and prolonged exposure to oxidative stress would lead to the pathophysiology of diseases such as diabetes, inflammation, and neurological disorders [52]. Natural antioxidants in food and beverage, e.g., tea, fruit, and vegetables, could react with free radicals and thus provide protection from age-related degenerative diseases [53,54]. Chiu et al. studied the antioxidant efficacy of protein-rich pearl powder in a randomized placebo-controlled trial with 20 participants [3]. The abundance of protein in pearl powder imparted its overall antioxidant activity. The protein-rich of pearl powder could substantially extend the lifespan of *Caenorhabditis elegans* due to its antioxidant activity. Both in vivo and in vitro studies indicated that pearl powder is an effective antioxidant and could potentially be used to treat multiple age-related degenerative diseases. Shao et al. showed that pearl powder is effective in beauty treatment and resistant to aging. However, particle size may influence antioxidant activity. Ultra-nano and ultra-micro pearl powders showed better free radical scavenging effects than water-soluble pearl powder [55]. Yang et al. reported that pearl powder bestow antihemolytic and antioxidant activities to protect human erythrocytes from 2,2’-azobis(2-amidinopropane) dihydrochloride (AAPH), which induces oxidative stress and causes damage to membrane proteins/lipids with concomitantly reduced hemolysis [2]. The protection of erythrocytes afforded by pearl powder is worthy of further molecular studies in elucidating the mechanism of action involved to help to discover new and effective therapies against diseases [2]. Getting a glass skin is a goal of new facial cosmetic goods. A recent randomized, placebo-controlled experiment on the use of blue pearl pigment reported that it could generate the perception of blue light effect, contributing to the transparency and gloss on Korean women’s faces [56].

### 3.4. Other Potential Health Effects

Pearl powder is a beneficial source of calcium [57]. Xia et al. investigated if pearl could help prevent cognitive morbidity and improve the metabolic processes of the hippocampus [58]. Insomnia-induced cognitive deficit was improved in both pearl-powder- and estradiol-treated rats relative to sleep-deprived rats. Proteomic results, however, suggested that the pharmacological effect on the gene expression induced by pearl powder and estradiol were different; pearl powder was more effective as insomnia treatment as it fixed more of the aberrant gene expression induced by sleep deprivation. Taken together with protein–protein interaction analysis, several pathways disrupted by sleep deprivation could be recovered by pearl powder treatment, including the retrograde endocannabinoid signalling pathway and protein interactions implicated in Parkinson’s and Huntington’s diseases. Chen et al. evaluated the calcium bioavailability of pearl powder of different sizes and found that nanoscale pearl powder improves calcium bioavailability [57], suggesting that nanoscale pearl powder may have better bioactivity compared to that of larger particle size.

### 3.5. Scaffolds for Tissue Engineering

Bone tissue engineering using osteoinductive scaffolds is an emerging approach to bone regeneration. Pearl possesses a unique block-and-mortar hierarchical structure and is potentially a good bone repair material as it has a higher osteogenic activity than HA or nacre [59]. At present, it still has limited use in therapeutic bone repair and regeneration. Electrospun fibres possess a three-dimensional (3D) structure that bears resemblance to the structure of bone ECM. These polymer scaffolds are usually made from components that have both bioactivity and mechanical strength optimized [60]. They can be combined with HA, Ca_3_(PO_4_)_2_ or calcium sulphate (CaSO_4_) which have good biocompatibility but low osteogenic activity [61]. In one of the earliest investigations, HA was shown to deposit on the surface of a pearl in simulated body fluid (SBF) via a dissolution-binding-precipitation mechanism [62]. High-resolution transmission electron microscopy (HR-TEM) images revealed that HA control was poorly crystallized and/or amorphous. As such, pearl powder can be included in the matrix of a biocompatible polymer as a bioactive particle filler that can promote bone growth while strengthen the composite [63,64]. Nacre has been shown to promote osteoblast proliferation and differentiation [62,65]. The biocompatibility, biodegradability, and osteogenic properties of nacre make it a promising bioactive filler for bone implants [66]. Lamghari et al. reported that nacre induced bone growth in vitro and in vivo [67]. Asvanund et al. observed that pearl promoted osteogenic differentiation of human bone cells as a result of increased ALP and osteocalcin (OCN) expression in vitro [68]. These authors suggested that nacre may contain signalling molecules that stimulate osteogenesis. For further details on nacre not covered herein, readers are referred to a comprehensive review on nacre previously published by Gerhard et al. [69].

**Figure 4:**
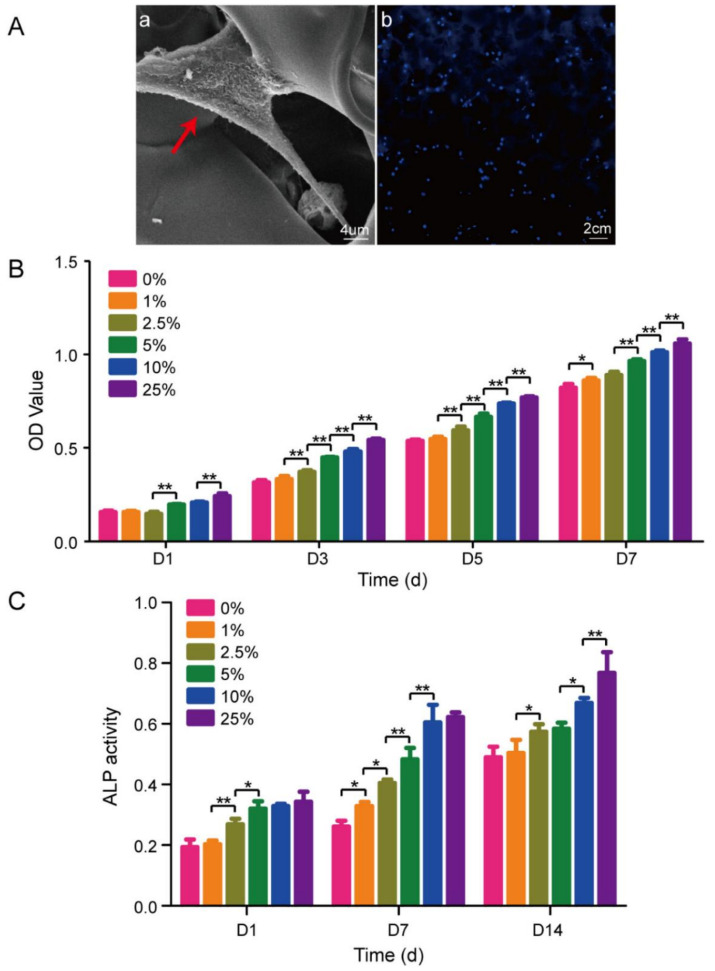
(**A**) SEM images: (a) cell morphology and (b) distribution of cells cultured on C-HLA/NPP scaffold after 4 weeks; (**B**) cell proliferation on scaffolds at day 1, 3, 5, and 7; (**C**) ALP activity of cells on day 1, 7, and 14. Reproduced from [70]. * *p* < 0.05, and ** *p* < 0.01.

Freeze-drying of chitosan-hyaluronic acid (C-HLA) with nano-pearl powder (NPP) produced a scaffold that successfully imitated the composition and structure of bone [70]. The hydrophilicity and mechanical strength of the scaffold improved with NPP content, but at the expense of pore morphology. Cell culture tests showed that scaffolds with a higher proportion of NPP (10–25 wt.%) efficiently promoted proliferation and differentiation of MC3T3-E1 cells (Figure 4A). In detail, cells entered an exponential growth phase after 3 days and proliferated at a much faster rate on scaffolds with NPP compared to non-NPP scaffolds (Figure 4B). ALP activity was greater for scaffolds with higher proportions of NPP (Figure 4C). It was found that cell differentiation was facilitated by controlled expression of genes COL-1, OCN, osteopontin (OPN) and RUNX2. 

**Figure 5:**
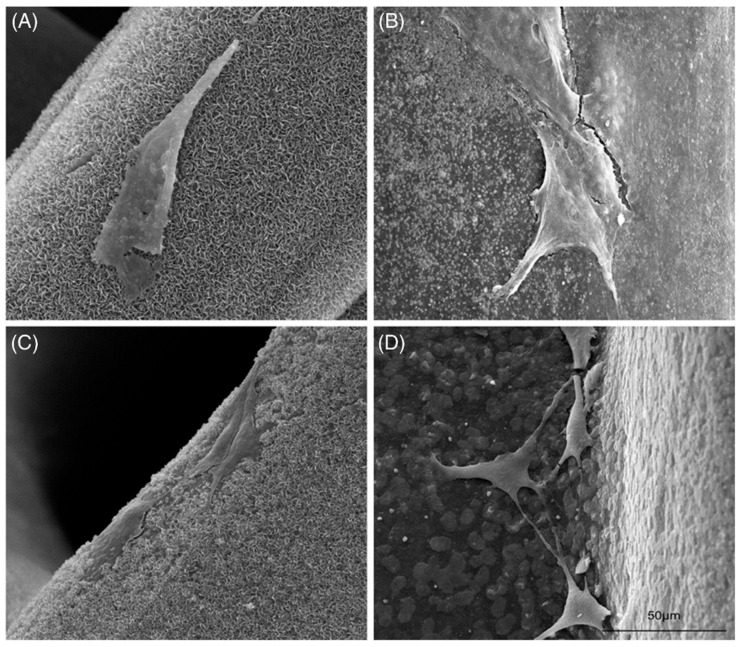
SEM images of adhered cells on (**A**) PCL, (**B**) 30 wt.% pearl/PCL, (**C**) 50 wt.% pearl/PCL (**D**) 80 wt.% pearl/PCL scaffolds, and (**D**) scaffolds after cultured for 7 days (scale bar: 50 μm). Reproduced from [71].

Zhang et al. fabricated PCL composite scaffolds with various concentrations of pearl powder (30–80 wt.%) with 3D printing [71]. The pore size and morphology of the scaffolds were tightly regulated by 3D printing, which would affect tissue growth and nutrients transfer. Standardized square macropores allow favourable mechanical strength. When the concentration of pearl powder within the scaffold increased, the compressive intensity and apatite formation increased, as did the cell adhesion, proliferation, and osteogenic differentiation (Figure 5). In brief, the physicochemical and biological properties of pearl powder/PCL composite scaffolds were strongly correlated with the concentration of pearl powder. 

Dai et al. impregnated natural pearl powder into polylactic acid (PLA) scaffold and observed that HA deposition on PLA/pearl composite increased by up to 15 wt.%, particularly at the beginning of mineralization [72], suggesting that a PLA/pearl powder scaffold could be more beneficial for bone repair than PLA alone. In a related work, pearl powder was integrated into an electrospun PLA scaffold [73]. The morphology study indicated that pearl powder was dispersed homogeneously on the surface of the PLA nanofibers, increasing their hydrophilicity. In vitro testing revealed PLA/pearl nanofibrous scaffold had higher cell proliferation and improved adhesion morphology of MC3T3 cells relative to PLA. A similar study compared poly-L-lactide (PLLA)/aragonite pearl powder, PLLA/vaterite pearl powder and PLLA/nacre powder scaffolds formed via freeze-drying [74]. SEM images indicated that the inclusion of powder did not make any detectable difference on the morphology of the composite scaffolds. All three scaffolds had approximately twice the compressive strength and modulus of pristine PLLA scaffold. PLLA/aragonite and PLLA/nacre scaffolds promoted greater cell proliferation of rat bone marrow-derived mesenchymal stem cells (rBMSCs) and ALP activity than pristine PLLA scaffold. However, PLLA/vaterite scaffold decreased the proliferation of rBMSC cells and osteogenic separation, and this is likely to be attributed to its elevated pH. It is worth noting that PLLA/vaterite and PLLA/aragonite scaffolds had a slower degradation rate compared to pristine PLLA [75]. Interestingly, an increase in pH of composite scaffolds can reduce the rate of hydrolysis of PLLA, demonstrating that pearl powder may have great potential in regulating the biodegradability of scaffolds.

In the work reported by Bai et al., pearl powder was mixed with poly(3-hydroxybutyrate-co-3-hydroxyvalerate) (PHBV) and a composite nanofiber scaffold was prepared from the mixture via electrospinning [76]. Mineralization on these composite nanofibres improved with an increasing proportion of pearl powder. Polyaminoacid (PAA) scaffolds impregnated with pearl powder have also been investigated as composites for bone healing [77]. These composites were prepared using an in situ melting polycondensation process to incorporate the high osteogenic influence of pearl with the flexibility and biocompatibility of PAA. This scaffold possessed superior mechanical properties, higher bioactivity, and osteogenic activity relative to PAA. Apatite particles were successfully deposited from SBF on the composite surface after 7 days. Pearl/PAA composite displayed improved mineralization, higher cell proliferation, and enhanced mouse bone marrow mesenchymal stem cell (MSCs) adhesion. In addition, cells that developed on the composite surface displayed higher ALP activity, calcified nodule production and expression of osteogenic differentiation-related genes (COL 1, RUNX2, OCN, and OPN) than cells developed on pristine PAA. 

**Figure 6:**
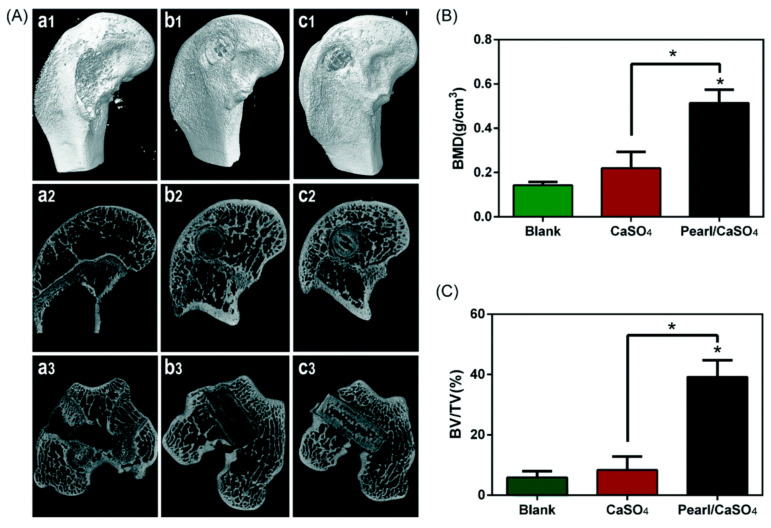
Post-implantation evaluation of repaired skulls. (**A**) 3D images of (a1–a3) control, (b1–b3) scaffolds from CaSO_4_, and (c1–c3) scaffolds from pearl/CaSO_4_; morphometric analysis of (**B**) bone mineral density, and (**C**) bone volume/total volume. Reproduced from [79]. * *p* < 0.01.

In an earlier study, natural pearl powders were used as fillers for the preparation of modified polypropylene (PP) monofilaments to enhance the biocompatibility of mesh used in pelvic reconstruction [78]. SEM images revealed masses of pearl powder on the surface of the monofilaments favoured cell proliferation and growth of L929 and PIEC cells. Du et al. fabricated pearl/CaSO_4_ composite scaffolds via 3D printing accompanied by a hydration process [79]. There are different views on the utility of CaSO_4_ as a biomaterial [80]. Pearl powder is predominately CaCO_3_ which buffers the pH of bone growth sites [81]. Pearl/CaSO_4_ scaffolds displayed uniform intertwined macropores (~400 mm) with high porosity. The scaffolds also exhibited improved compressive strength (~8 MPa), which is closely comparable to that of natural bone and twice the strength of a plaster-of-Paris, CaSO_4_ scaffold (∼3 MPa). These pearl/CaSO_4_ scaffolds possess strong apatite-forming potential, promoted the spread and differentiation of rBMSCs, and enhanced expression of associated osteogenic genes. Notably, the micro-computed tomography and histology of essential rabbit femoral condyle defects implanted with these scaffolds indicated osteogenic potential with fresh bone found after 8 weeks (Figure 6).

The use of resorbable devices for osteosynthesis is an emerging area of focus. Nacre was shown to support bone apposition without triggering an inflammatory response [82]. It was also observed that giant cells and osteoclasts were present in bone grafted with nacre [83]. These studies have demonstrated the promising potential of using nacre as a resorbable and osteoconductive material in the future.

Drug-charged implantable scaffolds can potentially be used for bone cancer chemotherapy, in combination with surgery [84]. Electrospun PLA/pearl powder nanofibers were impregnated with doxorubicin hydrochloride (DOX), yielding a drug-loaded nanofibrous scaffold (DOX@PLA/pearl) [85]. In vitro drug delivery study revealed that the distribution of DOX increased as the pearl content increased, as did the anti-tumour efficacy against HeLa cells, having great potential as an implantable scaffold for postoperative cancer treatment.

## 4. Concluding Remarks

The use of mother of pearl and pearl powder in traditional Chinese medicine as a cosmetic additive or food ingredient has a long and rich history. In this paper, we have briefly discussed the opportunities, developments and challenges in applications of the use of pearl powder in the treatment of various biomedical issues. Contemporary research of these natural materials has identified their potential biomedical applications with encouraging preliminary findings. Much more study is needed to delineate the possible attributes and mechanism of action of pearl powder for skincare and in medicine. However, a fundamental challenge to this development is that commercial pearl powder is often variable in its consistency and commonly adulterated with low-cost shell powder. Analytical methods are needed to differentiate pure pearl powder from adulterated material. Progress has been made in this respect using microscopic infrared reflectance spectroscopy. Given sufficient spectroscopic data, principal component analysis and partial least squares can provide confidence in distinguishing and categorizing pearl powder samples. This technological progress will be particularly important for the implantation of pearl powder in biomedical applications. As there is no formal attempt has been done to standardise the analytical protocols for the quality assessment of pearl powder among the manufacturers and researchers working on this subject, the meaningful comparison of data becomes extremely difficult. Even most fundamental characteristics of pearl powder can be determined by conventional analytical methods, the correlations between these methods and characteristics of pearl powder are poorly understood. In brief, pearl powder shows promise as an additive for wound healing and tissue engineering to promote cell growth on polymer scaffolds, but reliable identification, quantification and characterization of this complex mixture of components are important to gain a better understanding of its biochemical mechanism of action and expand its potential in biomedical application. Many questions and issues remain open and stimulate future research and innovative solutions. By combining the advantages offered by conventional techniques with the versatility of new processing techniques, new ways for scaling pearl powder production can be envisaged. Nevertheless, a substantial effort is required to eventually translate the exciting properties of pearl powder into a technological and commercial reality.

## Data Availability

Not Applicable.

## Abbreviations

ALP	alkaline phosphatase
Ca_3_(PO_4_)_2_	calcium phosphate
CaCO_3_	calcium carbonate
CaSO_4_	calcium sulphate
CCK-8	cell count kit-8
DOX	doxorubicin hydrochloride
ECM	extracellular matrix
ERK	extracellular signal-controlled kinase
FDA	Food and Drug Administration
HA	hydroxyapatite
MEK	mitogen-activated protein kinase
NPP	nano-pearl powder
PAA	polyaminoacid
PCL	polycaprolactone
PDGF	platelet-derived growth factor
PHBV)	poly(3-hydroxybutyrate-co-3-hydroxyvalerate
PLA	polylactic acid
PLLA	poly-L-lactide
PP	polypropylene
rBMSC	rat bone marrow-derived mesenchymal stem cells
ROS	reactive oxygen species
RUNX2	runt-related transcription factor 2
SBF	simulated body fluid
SPP1	secreted phosphoprotein l
WSM	water-soluble matrix

## References

[1] Zhang J., Li S., Yao S., Si W., Cai L., Pan H., Hou J., Yang W., Da J., Jiang B. (2015). Ultra-performance liquid chromatography of amino acids for the quality assessment of pearl powder. J. Sep. Sci..

[2] Yang H.-L., Korivi M., Lin M.-K., Chang H.C.-W., Wu C.-R., Lee M.-S., Chen W.T.-L., Hseu Y.-C. (2017). Antihemolytic and antioxidant properties of pearl powder against 2,2′-azobis(2-amidinopropane) dihydrochloride-induced hemolysis and oxidative damage to erythrocyte membrane lipids and proteins. J. Food Drug Anal..

[3] Chiu H.-F., Hsiao S.-C., Lu Y.-Y., Han Y.-C., Shen Y.-C., Venkatakrishnan K., Wang C.-K. (2018). Efficacy of protein rich pearl powder on antioxidant status in a randomized placebo-controlled trial. J. Food Drug Anal..

[4] Chen X., Peng L.-H., Chee S.-S., Shan Y.-H., Liang W.-Q., Gao J.-Q. (2019). Nanoscaled pearl powder accelerates wound repair and regeneration in vitro and in vivo. Drug Dev. Ind. Pharm..

[5] Nagai K. (2013). A history of the cultured pearl industry. Zool. Sci..

[6] Yu Z.R., Wang X.D., Su B.M., Zhang Y. (2017). First evidence of the use of freshwater pearls as a cosmetic in ancient China: Analysis of white makeup powder from a Northern Song dynasty Lv tomb (Lantian, Shaanxi province, China). Archaeometry.

[7] Latire T., Legendre F., Bouyoucef M., Marin F., Carreiras F., Rigot-Jolivet M., Lebel J.-M., Galéra P., Serpentini A. (2017). Shell extracts of the edible mussel and oyster induce an enhancement of the catabolic pathway of human skin fibroblasts, in vitro. Cytotechnology.

[8] Lee K., Kim H., Kim J.M., Chung Y.H., Lee T.Y., Lim H.-S., Lim J.-H., Kim T., Bae J.S., Woo C.-H. (2012). Nacre-driven water-soluble factors promote wound healing of the deep burn porcine skin by recovering angiogenesis and fibroblast function. Mol. Biol. Rep..

[9] Li Y.-C., Chen C.-R., Young T.-H. (2013). Pearl extract enhances the migratory ability of fibroblasts in a wound healing model. Pharm. Biol..

[10] Werner S., Krieg T., Smola H. (2007). Keratinocyte–fibroblast interactions in wound healing. J. Investig. Dermatol..

[11] Gröber U., Schmidt J., Kisters K. (2015). Magnesium in prevention and therapy. Nutrients.

[12] Tsukamoto D., Sarashina I., Endo K. (2004). Structure and expression of an unusually acidic matrix protein of pearl oyster shells. Biochem. Biophys. Res. Commun..

[13] Zhang C., Xie L., Huang J., Liu X., Zhang R. (2006). A novel matrix protein family participating in the prismatic layer framework formation of pearl oyster, *Pinctada fucata*. Biochem. Biophys. Res. Commun..

[14] Park S.Y., Ahn C.-B., Je J.-Y. (2014). Antioxidant and anti-inflammatory activities of protein hydrolysates from mytilus edulis and ultrafiltration membrane fractions. J. Food Biochem..

[15] Saiga A., Tanabe S., Nishimura T. (2003). Antioxidant activity of peptides obtained from porcine myofibrillar proteins by protease treatment. J. Agric. Food Chem..

[16] Ren J., Zhao M., Shi J., Wang J., Jiang Y., Cui C., Kakuda Y., Xue S.J. (2008). Purification and identification of antioxidant peptides from grass carp muscle hydrolysates by consecutive chromatography and electrospray ionization-mass spectrometry. Food Chem..

[17] Iranzo O. (2011). Manganese complexes displaying superoxide dismutase activity: A balance between different factors. Bioorg. Chem..

[18] Westbroek P., Marin F. (1998). A marriage of bone and nacre. Nature.

[19] Rousseau M., Pereira-Mouriès L., Almeida M.-J., Milet C., Lopez E. (2003). The water-soluble matrix fraction from the nacre of *Pinctada maxima* produces earlier mineralization of MC3T3-E1 mouse pre-osteoblasts. Comp. Biochem. Physiol. Part B Biochem. Mol. Biol..

[20] Lakshminarayanan R., Chi-Jin E.O., Loh X.J., Kini R.M., Valiyaveettil S. (2005). Purification and Characterization of a Vaterite-Inducing Peptide, Pelovaterin, from the Eggshells of Pelodiscus sinensis (Chinese Soft-Shelled Turtle). Biomacromolecules.

[21] Lakshminarayanan R., Loh X.J., Gayathri S., Sindhu S., Banerjee Y., Kini R.M., Valiyaveettil S. (2006). Formation of Transient Amorphous Calcium Carbonate Precursor in Quail Eggshell Mineralization:  An In Vitro Study. Biomacromolecules.

[22] Wang L., Sondi I., Matijević E. (1999). Preparation of Uniform Needle-Like Aragonite Particles by Homogeneous Precipitation. J. Colloid Interface Sci..

[23] Nan Z., Chen X., Yang Q., Wang X., Shi Z., Hou W. (2008). Structure transition from aragonite to vaterite and calcite by the assistance of SDBS. J. Colloid Interface Sci..

[24] Vecht A., Ireland T.G. (2000). The role of vaterite and aragonite in the formation of pseudo-biogenic carbonate structures: Implications for Martian exobiology. Geochim. Cosmochim. Acta.

[25] Grasby S.E. (2003). Naturally precipitating vaterite (μ-CaCO_3_) spheres: Unusual carbonates formed in an extreme environment. Geochim. Cosmochim. Acta.

[26] Fan Y.W., Wang R.Z. (2005). Submicrometer-Sized Vaterite Tubes Formed through Nanobubble-Templated Crystal Growth. Adv. Mater..

[27] Ma Y., Gao Y., Feng Q. (2011). Characterization of organic matrix extracted from fresh water pearls. Mater. Sci. Eng. C.

[28] Ma Y.F., Gao Y.H., Feng Q.L. (2010). Effects of pH and temperature on CaCO_3_ crystallization in aqueous solution with water soluble matrix of pearls. J. Cryst. Growth.

[29] Miyamoto H., Miyashita T., Okushima M., Nakano S., Morita T., Matsushiro A. (1996). A carbonic anhydrase from the nacreous layer in oyster pearls. Proc. Natl. Acad. Sci. USA.

[30] Samata T., Hayashi N., Kono M., Hasegawa K., Horita C., Akera S. (1999). A new matrix protein family related to the nacreous layer formation of *Pinctada fucata*. FEBS Lett..

[31] Miyashita T., Takagi R., Okushima M., Nakano S., Miyamoto H., Nishikawa E., Matsushiro A. (2000). Complementary DNA Cloning and Characterization of Pearlin, a New Class of Matrix Protein in the Nacreous Layer of Oyster Pearls. Mar. Biotechnol..

[32] Michenfelder M., Fu G., Lawrence C., Weaver J.C., Wustman B.A., Taranto L., Evans J.S., Morse D.E. (2003). Characterization of two molluscan crystal-modulating biomineralization proteins and identification of putative mineral binding domains. Biopolymers.

[33] Kim I.W., Collino S., Morse D.E., Evans J.S. (2006). A Crystal Modulating Protein from Molluscan Nacre That Limits the Growth of Calcite in Vitro. Cryst. Growth Des..

[34] Gong N., Li Q., Huang J., Fang Z., Zhang G., Xie L., Zhang R. (2008). Culture of outer epithelial cells from mantle tissue to study shell matrix protein secretion for biomineralization. Cell Tissue Res..

[35] Zhang C., Li S., Ma Z., Xie L., Zhang R. (2006). A Novel Matrix Protein p10 from the Nacre of Pearl Oyster (*Pinctada fucata*) and Its Effects on Both CaCO_3_ Crystal Formation and Mineralogenic Cells. Mar. Biotechnol..

[36] Pereira-Mouriès L., Almeida M.-J., Ribeiro C., Peduzzi J., Barthélemy M., Milet C., Lopez E. (2002). Soluble silk-like organic matrix in the nacreous layer of the bivalve *Pinctada maxima*. Eur. J. Biochem..

[37] Aizenberg J., Hanson J., Koetzle T.F., Weiner S., Addadi L. (1997). Control of Macromolecule Distribution within Synthetic and Biogenic Single Calcite Crystals. J. Am. Chem. Soc..

[38] Zhang X., Hu C., Yan Y., Yang H.F., Li J.F., Bai H., Xi G.C., Liao J. (2014). Identification of Pearl Powder Using Microscopic Infrared Reflectance Spectroscopy. Spectrosc. Spectr. Anal..

[39] Liu S.Q., Wei W., Bai Z.Y., Wang X.C., Li X.H., Wang C.X., Liu X., Liu Y., Xu C.H. (2018). Rapid identification of pearl powder from Hyriopsis cumingii by Tri-step infrared spectroscopy combined with computer vision technology. Spectroc. Acta Part A Mol. Biomol. Spectr..

[40] Dhand C., Venkatesh M., Barathi V.A., Harini S., Bairagi S., Goh Tze Leng E., Muruganandham N., Low K.Z.W., Fazil M.H.U.T., Loh X.J. (2017). Bio-inspired crosslinking and matrix-drug interactions for advanced wound dressings with long-term antimicrobial activity. Biomaterials.

[41] Opalenik S.R., Davidson J.M. (2005). Fibroblast differentiation of bone marrow-derived cells during wound repair. FASEB J..

[42] Rhett J.M., Ghatnekar G.S., Palatinus J.A., O’Quinn M., Yost M.J., Gourdie R.G. (2008). Novel therapies for scar reduction and regenerative healing of skin wounds. Trends Biotechnol..

[43] Fox S.J., Fazil M.H.U.T., Dhand C., Venkatesh M., Goh E.T.L., Harini S., Eugene C., Lim R.R., Ramakrishna S., Chaurasia S.S. (2016). Insight into membrane selectivity of linear and branched polyethylenimines and their potential as biocides for advanced wound dressings. Acta Biomater..

[44] Mayandi V., Wen Choong A.C., Dhand C., Lim F.P., Aung T.T., Sriram H., Dwivedi N., Periayah M.H., Sridhar S., Fazil M.H.U.T. (2020). Multifunctional Antimicrobial Nanofiber Dressings Containing ε-Polylysine for the Eradication of Bacterial Bioburden and Promotion of Wound Healing in Critically Colonized Wounds. ACS Appl. Mater. Interfaces.

[45] Neri S., Miyashita T., Hashimoto H., Suda Y., Ishibashi M., Kii H., Watanabe H., Kuwata T., Tsuboi M., Goto K. (2017). Fibroblast-led cancer cell invasion is activated by epithelial–mesenchymal transition through platelet-derived growth factor BB secretion of lung adenocarcinoma. Cancer Lett..

[46] Lopez E., Faou A.L., Borzeix S., Berland S. (2000). Stimulation of rat cutaneous fibroblasts and their synthetic activity by implants of powdered nacre (mother of pearl). Tissue Cell.

[47] Rousseau M., Bédouet L., Lati E., Gasser P., Le Ny K., Lopez E. (2006). Restoration of stratum corneum with nacre lipids. Comp. Biochem. Physiol. Part B Biochem. Mol. Biol..

[48] Tanaka S., Hatano H., Itasaka O. (1960). Biochemical Studies on Pearl. IX. Amino Acid Composition of Conchiolin in Pearl and Shell. Bull. Chem. Soc. Jpn..

[49] Jian-Ping D., Jun C., Yu-Fei B., Bang-Xing H., Shang-Bin G., Li-Li J. (2010). Effects of pearl powder extract and its fractions on fibroblast function relevant to wound repair. Pharm. Biol..

[50] Cheng Y.A., Zhang W.B., Fan H., Xu P. (2018). Water-soluble nano-pearl powder promotes MC3T3-E1 cell differentiation by enhancing autophagy via the MEK/ERK signaling pathway. Mol. Med. Rep..

[51] Yang Y.-L., Chang C.-H., Huang C.-C., Liu H.-W. (2015). Anti-inflammation and anti-apoptosis effects of pearl extract gel on UVB irradiation HaCaT cells. Bio-Med. Mater. Eng..

[52] Zhao G.-R., Xiang Z.-J., Ye T.-X., Yuan Y.-J., Guo Z.-X. (2006). Antioxidant activities of Salvia miltiorrhiza and Panax notoginseng. Food Chem..

[53] Hsieh C.-C., Liao C.-C., Liao Y.-C., Hwang L.S., Wu L.-Y., Hsieh S.-C. (2016). Proteomic changes associated with metabolic syndrome in a fructose-fed rat model. J. Food Drug Anal..

[54] Havsteen B.H. (2002). The biochemistry and medical significance of the flavonoids. Pharmacol. Ther..

[55] Shao D.-Z., Wang C.-K., Hwang H.-J., Hung C.-H., Chen Y.-W. (2010). Abstracts: Comparison of hydration, tyrosinase resistance, and antioxidant activation in three kinds of pearl powders. Int. J. Cosmet. Sci..

[56] Lee M., Park S.-J., Jeong C., Jang S.I., Han J., Kim B.J., Kim E. (2020). Perception of the blue light effect on Korean women’s faces using the blue pearl pigment. Ski. Res. Technol..

[57] Chen H.S., Chang J.H., Wu J.S.B. (2008). Calcium bioavailability of nanonized pearl powder for adults. J. Food Sci..

[58] Xia M., Huang D.L., Tong Y.M., Lin J. (2020). Pearl powder reduces sleep disturbance stress response through regulating proteomics in a rat model of sleep deprivation. J. Cell. Mol. Med..

[59] Jarcho M. (1981). Calcium phosphate ceramics as hard tissue prosthetics. Clin. Orthop. Relat. Res..

[60] Yu X., Ibrahim M., Liu Z., Yang H., Tan L., Yang K. (2018). Biofunctional Mg coating on PEEK for improving bioactivity. Bioact. Mater..

[61] Ning C.Q., Zhou Y. (2002). In vitro bioactivity of a biocomposite fabricated from HA and Ti powders by powder metallurgy method. Biomaterials.

[62] Shen Y., Zhu J., Zhang H., Zhao F. (2006). In vitro osteogenetic activity of pearl. Biomaterials.

[63] Liu Y.S., Huang Q.L., Feng Q.L., Hu N.M., Albert O. (2013). Structural features and mechanical properties of PLLA/pearl powder scaffolds. J. Mech. Med. Biol..

[64] Shuai C., Guo W., Gao C., Yang Y., Xu Y., Liu L., Qin T., Sun H., Yang S., Feng P. (2017). Calcium silicate improved bioactivity and mechanical properties of poly(3-hydroxybutyrate-co-3-hydroxyvalerate) scaffolds. Polymers.

[65] Pereira Mouriès L., Almeida M.-J., Milet C., Berland S., Lopez E. (2002). Bioactivity of nacre water-soluble organic matrix from the bivalve mollusk *Pinctada maxima* in three mammalian cell types: Fibroblasts, bone marrow stromal cells and osteoblasts. Comp. Biochem. Physiol. Part B Biochem. Mol. Biol..

[66] Flausse A., Henrionnet C., Dossot M., Dumas D., Hupont S., Pinzano A., Mainard D., Galois L., Magdalou J., Lopez E. (2013). Osteogenic differentiation of human bone marrow mesenchymal stem cells in hydrogel containing nacre powder. J. Biomed. Mater. Res. Part A.

[67] Lamghari M., Almeida M.J., Berland S., Huet H., Laurent A., Milet C., Lopez E. (1999). Stimulation of bone marrow cells and bone formation by nacre: In vivo and in vitro studies. Bone.

[68] Asvanund P., Chunhabundit P., Suddhasthira T. (2011). Potential induction of bone regeneration by nacre: An in vitro study. Implant Dent..

[69] Gerhard E.M., Wang W., Li C., Guo J., Ozbolat I.T., Rahn K.M., Armstrong A.D., Xia J., Qian G., Yang J. (2017). Design strategies and applications of nacre-based biomaterials. Acta Biomater..

[70] Li X.N., Xu P., Cheng Y.N., Zhang W.B., Zheng B., Wang Q.Z. (2020). Nano-pearl powder/chitosan-hyaluronic acid porous composite scaffold and preliminary study of its osteogenesis mechanism. Mater. Sci. Eng. C Mater. Biol. Appl..

[71] Zhang X., Du X.Y., Li D.J., Ao R.G., Yu B., Yu B.Q. (2018). Three Dimensionally Printed Pearl Powder/Poly-Caprolactone Composite Scaffolds for Bone Regeneration. J. Biomater. Sci. Polym. Ed..

[72] Dai J.M., Bai J.J., Jin J.H., Yang S.L., Li G. (2015). Stimulation by pearl of mineralization and biocompatibility of PLA. Adv. Eng. Mater..

[73] Dai J.M., Yang S.L., Jin J.H., Li G. (2016). Electrospinning of PLA/pearl powder nanofibrous scaffold for bone tissue engineering. RSC Adv..

[74] Liu Y.S., Huang Q.L., Feng Q.L. (2013). 3D scaffold of PLLA/pearl and PLLA/nacre powder for bone regeneration. Biomed. Mater..

[75] Liu Y.S., Huang Q.L., Kienzle A., Müller W.E.G., Feng Q.L. (2014). In vitro degradation of porous PLLA/pearl powder composite scaffolds. Mater. Sci. Eng. C.

[76] Bai J.J., Dai J.M., Li G. (2015). Electrospun composites of phbv/pearl powder for bone repairing. Prog. Nat. Sci..

[77] Wu Y.N., Ding Z.W., Ren H.H., Ji M.Z., Yan Y.G. (2019). Preparation, Characterization and In Vitro Biological Evaluation of a Novel Pearl Powder/Poly-Amino Acid Composite as a Potential Substitute for Bone Repair and Reconstruction. Polymers.

[78] Deng Y.H., Li G., Song W.L., Jiang J.M. (2015). Preparation and properties of pearl powder/polypropylene composites and their biocompatibility. Bio-Med. Mater. Eng..

[79] Du X.Y., Yu B., Pei P., Ding H.F., Yu B.Q., Zhu Y.F. (2018). 3D printing of pearl/CaSO_4_ composite scaffolds for bone regeneration. J. Mat. Chem. B.

[80] Ding Y., Tang S., Yu B., Yan Y., Li H., Wei J., Su J. (2015). In vitro degradability, bioactivity and primary cell responses to bone cements containing mesoporous magnesium–calcium silicate and calcium sulfate for bone regeneration. J. R. Soc. Interface.

[81] Horie M., Nishio K., Kato H., Endoh S., Fujita K., Nakamura A., Kinugasa S., Hagihara Y., Yoshida Y., Iwahashi H. (2014). Evaluation of cellular influences caused by calcium carbonate nanoparticles. Chem. Biol. Interact..

[82] Kün-Darbois J.-D., Libouban H., Camprasse G., Camprasse S., Chappard D. (2020). In vivo osseointegration and erosion of nacre screws in an animal model. J. Biomed. Mater. Res. Part B Appl. Biomater..

[83] Chappard D., Kün-Darbois J.-D., Pascaretti-Grizon F., Camprasse G., Camprasse S. (2019). Giant cells and osteoclasts present in bone grafted with nacre differ by nuclear cytometry evaluated by texture analysis. J. Mater. Sci. Mater. Med..

[84] Li Z., Loh X.J. (2017). Recent advances of using polyhydroxyalkanoate-based nanovehicles as therapeutic delivery carriers. Wires Nanomed. Nanobiotechnol..

[85] Dai J.M., Jin J.H., Yang S.L., Li G. (2017). Doxorubicin-loaded PLA/pearl electrospun nanofibrous scaffold for drug delivery and tumor cell treatment. Mater. Res. Express.

